# Insanity of Advanced Life
*A Practical Treatise on the Domestic Management and most important Diseases of Advanced Life, by George E. Day, M.D., Fellow of the Royal College of Physicians, &c. T. and W. Boone, London, 1849. pp. 342.


**Published:** 1850-04-01

**Authors:** 


### ART. V.—INSANITY OF ADVANCED LIFE.*

GREY hairs and senility always command respect, even from the most thoughtless; but when in old age there is also mental vigour, no one can look upon the possessor of such qualifications without feelings of great veneration. But many exceptions to this venerable condition, alas! prevail; and as the physical frame of man usually deteriorates with advancing age, the mental faculties, after the grand climacteric, then become so weakened, that individuals in their declining years of life sometimes are as imbecile in mind, as they were eminent for intellectual power at an early period of their

* A Practical Treatise on the Domestic Management and most important Diseases of Advanced Life, by George E. Day, M.D., Fellow of the Royal College of Physicians, &c. T. and W. Boone, London, 1849. pp. 342.

lives. Thus the great talents of a Marlborough seemed confounded in the latter years of his life, and his powerful mind became impaired; whilst in the same way, also, was extinguished the active spirit of the celebrated Dean Swift, as expressed in the often-quoted lines of Dr. Johnson.

> "From Marlbro's eyes the tears of dotage flow,
> And Swift expires a driveller and a show."

According to the experience of physicians, the intellectual faculties of man generally undergo decay, as the individual advances towards "the evening twilight of his existence." Amongst other authorities, Esquirol and Leuret may be mentioned, who have arrived at this conclusion; and from the evidence afforded by the examination of 12,869 cases, these writers think that the probability of insanity supervening, increases with advancing years. Other authors might be quoted in support of the above conclusion; but the observation of inquirers on this point coincides so generally in opinion, that farther illustration really seems superfluous.

In discussing the mental diseases of advanced life, the author of the work before us considers there are four forms which may be characterized as specially belonging to the period of life, of which his volume professedly treats. These varieties, according to Dr. Day, are—

"1. Insanity connected with the cessation of the menstrual discharge, with the suppression of hæmorrhages, or with the rapid healing of ulcers or skin diseases.

"2. Insanity depending probably on a diseased condition of the cerebellum, or of the prostate in men, or of the ovaries or uterus in women, and exhibiting itself in abnormal, or excessive erotic desires.

"3. Insanity usually commencing about the age of sixty, without reference to sex, and apparently connected pathologically with the altered character of the cerebral circulation, that generally supervenes about that period.

"4. Senile dementia or fatuity."

Respecting "the abnormal erotic propensities" alluded to in the second division, one of our most philosophic and able writers on insanity, and whose recent death science and humanity so much deplore, namely, the late Dr. Pritchard, says, "have given rise to a series of phenomena in human actions, which have been considered to belong to the province of the moralist, or the enactor of penal chastisements, rather than to that of the medical philosopher. That this opinion has been founded in error we are fully convinced, and we doubt not that the time will come, when the very names of many offences against decorum, now considered punishable crimes, will be erased from the statute-book; and when persons, now liable to be sentenced to the pillory or the gallows, will be treated as lunatics."

The treatment of cases comprised within this category is sufficiently obvious, if the cause of the irritation can be clearly ascertained, although it too often happens that the disease is overlooked or neglected, at least, until the patient commits some gross outrage against public decorum, when his malady then becomes but too manifest to leave any doubt on the subject.

Respecting the third form enumerated by the author, and which has been admirably described by Dr. Seymour, in the first volume of his recent work, entitled "Thoughts on the Nature and Treatment of several severe Diseases," we would refer the reader to Dr. Day's description, contained in the following quotation:—

"The patient becomes morose, is exceedingly distressed and annoyed about trifles, and frequently has gloomy forebodings of the future. He suspects his old and best friends, fancies there are conspiracies against him, or has a morbid fear of dying a pauper. We must beware of suicide in these cases. I have been recently consulted regarding the case of a rich old gentleman about seventy years of age, of sanguineous temperament and strong frame, who made a large fortune by his own exertions, and for the last six or seven years has retired from business. With no definite object or resource, he has spent his leisure days in pondering over the horrors of a speedy chartist rule in England, and this predominant idea is so strangely mixed up with so strong a feeling of the extreme necessity for economy, that although he would on no account dispense with a good dinner and the most expensive wines, there is the greatest difficulty in persuading him to *pay* for the most necessary articles of life. The smallest demand for money is instantly suggestive of the workhouse, which unfortunately for the poor old gentleman's happiness is actually visible from his library window.

"The treatment recommended consisted essentially in an increased amount of exercise in the open air, (chiefly horse exercise;) alternate cupping from the nape of the neck, and leeches to the anus at intervals of a month or six weeks; a sufficient dose of a mixture of the common black draught and compound decoction of aloes, to ensure at least two motions; and an opiate at bedtime.

"He has now been under this course of treatment for about two months, and I hear that his temper is much improved, that the moroseness and gloominess have altogether disappeared, and although the principal delusions are not altogether removed, that he regards the impending miseries of his country as a due and proper retribution, ordained by a wise Providence, for the passing of the Reform Bill. I entertain strong hopes of his further improvement."

In order to obtain a clear perception of the condition enumerated under the fourth head — viz., "Senile dementia or fatuity," the author briefly notices certain points connected with the ordinary state of the mind of old persons, in which he says:—

"The intellectual powers usually remain unimpaired longer than the physical. We find, however, that the mind (*the spiritual life* of Canstatt and other German writers,) seems rather to live on the matters hoarded up in past times, than to continue to absorb fresh nutriment through the external senses; for the organs of these senses have undergone physical alterations, and the impressions conveyed to the brain are, as it were, dimmed and blunted. Hence while his judgment and opinion on the things and times of his youth are usually correct, the old man is liable to arrive at very incorrect results regarding the things occurring around him. Whilst his remembrance of the acts of yesterday is blotted out, the recollections of his childhood and youth are strong and deep. Living almost solely in the reminiscences of the past, the present holds out no attractions for him. He feels that the years so graphically described in sacred writ have come, 'when thou shalt say, I have no pleasure in them,' when 'the grasshopper shall be a burden, and desire shall fail.'"

The gradual decline of the mental faculties, in so far as the change bears on the question under discussion, is then alluded to by the author; and, after mentioning the order in which memory is first affected, Dr. Day remarks, in the words of Dr. Holland:—

"The faculty of receiving and associating fresh impressions for the most part declines earlier than the power of combining and using those formerly received; and the faculty of directing and fixing combinations or successions of ideas, is one of those earliest impaired. In some cases, a word, or even part of a word, of double application, will suddenly and without the consciousness of change, carry off the mind to a new and wholly foreign subject; and in others, in which the memory is remarkably tenacious as to persons and detailed events of past life, there is a singular incapacity of associating them together by any reasonable link; and the slightest relations of time or place suffice to carry the mind wholly astray from its subject. In reference to the decay of memory, the power of recollecting words and names is lost earlier and more readily than that of events; the ideas are often sufficiently distinct, while the words required for their expression are either not forthcoming, or others in no way applicable are substituted."

Although senile dementia is not the universal fate of old persons, nevertheless there is often a general tendency towards that condition; and amongst the various causes which accelerate the supervention of this altered state in the intellectual faculties of old people, the author enumerates: "1, Prolonged mental exertion; 2, the too free use of wine or spirits; 3, venereal excesses."

This affection, likewise, often follows slight attacks of apoplexy, when its progress then becomes very rapid. In illustration of the third form, or that arising from venereal excesses, the author quotes an interesting case, first reported by Sir Alexander Crichton, which we also transfer to our pages, both as an instructive example of senile dementia and as a warning to all salaceous old gentlemen:—

"The first case of this kind which occurred to me in practice, was that of an attorney, much respected for his integrity and talents, but who had many sad failings to which our physical nature too often subjects us. Although nearly in his seventieth year, and married to an amiable lady, much younger than himself, he kept a mistress whom he was in the habit of visiting every evening. The arms of Venus are not wielded with impunity at the age of seventy. He was suddenly seized with a great prostration of strength, giddiness, forgetfulness, insensibility to all concerns of life, and every symptom of approaching fatuity. His forgetfulness was of the kind alluded to. When he wished to ask for anything, he constantly made use of some inappropriate term. Instead of asking for a piece of bread, he would probably ask for his boots; but if these were brought, he knew they did not correspond with the idea he had of the thing he wished to have, and was therefore angry; yet he would still demand some of his boots or shoes, meaning bread. If he wanted a tumbler to drink out of, it was a thousand to one, he did not call for a certain chamber utensil; and if it was the said utensil he wanted, he would call it a tumbler, or a dish. He evidently was conscious that he pronounced the wrong words, for when the proper expressions were spoken by another person, and he was asked if it was not such a thing he wanted, he always seemed aware of his mistake, and corrected himself, by adopting the appropriate expression. This gentleman was cured of his complaint by large doses of valerian, and other proper medicines."

Speaking generally, in reference to senile dementia, we would remark, that frequently the character and conduct of the patient is so much altered, that he often seems quite another being; the pious often becomes impious; the pleased and happy discontented or miserable; the economical and prudent, wasteful or profuse; the generous, miserly, and the sober, intemperate. Those in whom the sexual appetite has long remained dormant, sometimes suddenly become immodest, dissipated, and addicted to all kinds of debauchery. In short, the entire demeanour of the unfortunate sufferers is often so altered, that, instead of being, as heretofore, respected by friends and acquaintances for their correct conduct, they are now objects of pity and commiseration.

Respecting the management of mental diseases during advanced life, the author is very concise; indeed, when the complaint is once firmly established, very little can be accomplished; and he believes that all remedies will prove unavailing, or nearly useless. In the opinion of Dr. Day, "mild tonic treatment, with due attention to hygienic precautions, is all that can be suggested;" and this view certainly coincides with our own experience in similar maladies. The measures then employed must be generally purely palliative; and when no organic lesion apparently exists, producing this form of mental disease, if due care be taken to prevent the consequences of delirious paroxysms, should any supervene, appropriate moral and medical treatment will occasionally do much to ameliorate the symptoms, as likewise to improve the condition of the patient. As a general principle it may be laid down, that the physician should employ, in the malady under discussion, those remedies which will remove inordinate action in the patient's system, without reducing strength; at the same time that appropriate means are used to regulate the natural functions of the body, to renovate the constitution, and to occupy, as also to amuse, the afflicted sufferer whenever practicable.

Notwithstanding the existence of insanity in old people, of even the most incurable form, human life may be sometimes prolonged to a very advanced period. This fact is frequently recorded in the reports of lunatic asylums, and by medical authors; amongst whom we may refer to a valuable and interesting paper recently published in the 32nd volume of the "Transactions of the Royal Medical and Chirurgical Society," by Dr. Webster, in which, when alluding to the incurable patients in Bethlem Hospital, he speaks of the long period of time some of the patients continued insane. One female had been twenty-nine years a lunatic, another female nearly thirty years, and a third forty-two years in the same lamentable condition. But the most remarkable instance of long-continued lunacy, without any intermission, was a female patient who had been an inmate of Bethlem Hospital for not less than fifty-four consecutive years. This unfortunate and afflicted human being—deprived of reason—shut out from all worldly enjoyment, by her continuous residence in this lunatic asylum for upwards of half a century, and having farther a weak physical constitution, nevertheless attained an advanced age. Amongst male lunatics, fewer instances of long-continued insanity were recorded than amongst the insane females. However, two men had been insane, in both instances upwards of thirty years; whilst another male lunatic had resided in Bethlem Hospital for forty-two years continuously. The above cases at least prove that male, but especially female lunatics may live for a long period, notwithstanding the existence of their incurable mental malady.

Before taking leave of Dr. Day, whose work, irrespective of the section now more immediately passed under review, we recommend, not only to our readers, but to all engaged in the practice of medicine, knowing their time will be usefully occupied in its perusal, we would congratulate him on his recent appointment as Chandos Professor of Medicine in the ancient University of St. Andrew's; where, although a worthy successor of the late accomplished Dr. Reid, and now the colleague of Sir David Brewster, so distinguished in science, and of other able coadjutors, we have no doubt Dr Day will realize the expectations friends entertain of his future professional career. May success attend his labours, and we also trust his lot may be happy as well as prosperous.

---

